# Unusual presentation of gastric cancer during treatment of hairy cell leukemia: Exploring the etiological basis of this rare phenomenon

**DOI:** 10.1016/j.cpt.2023.01.003

**Published:** 2023-01-12

**Authors:** Shahan Tariq, Muhammad Ammar Bin Hamid, Nazia Rahman, Lindsey Oleary, Kristine Wong, Aasim Sehbai

**Affiliations:** a Department of Hematology/Oncology, Regional Medical Center (RMC), Anniston, AL, 36207, USA; b Department of Internal Medicine, University of South Dakota Sanford School of Medicine, Sioux Falls, SD, 57069, USA; c Alabama College of Osteopathic Medicine, Dothan, AL, 36303, USA

**Keywords:** Cystic lesion, Gastric cancer, Hairy cell leukemia

## Abstract

Hairy cell leukemia (HCL) is a rare B-cell lymphoproliferative disorder. Patients typically present with cytopenia and splenomegaly. We describe the case of a 78-year-old patient with refractory HCL who acutely developed a cystic lesion on the back while receiving moxetumomab pasudotox therapy. Biopsy of the lesion revealed the presence of adenocarcinoma, which prompted a detailed evaluation resulting in a diagnosis of stage IV gastric cancer. Nevertheless, to establish any association between moxetumomab pasudotox therapy and secondary cancer development, a satisfactory number of studies need to be conducted.

## Highlights

• A patient with hairy cell leukemia treated with moxetumomab pasudotox developed gastric cancer.
• The long-term side effects of moxetumomab pasudotox remain largely undocumented.
• Healthcare professionals should remain vigilant when prescribing moxetumomab.

## Introduction

Hairy cell leukemia (HCL) is a chronic B-cell lymphoproliferative disorder that accounts for approximately 3% of all adult leukemia in the United States.^1^ It usually presents as splenomegaly, infection, or decreased cell counts. For symptomatic cases, cladribine and pentostatin are first-line treatment modalities. Moxetumomab pasudotox-tdfk, a new recombinant immunotoxin, was approved for use in refractory or relapsing HCL.^1^ We discuss factors leading to the rare presentation of gastric cancer in a patient undergoing treatment for refractory HCL.

## Case presentation

A 78-year-old man, with refractory HCL was initially diagnosed in July 2018 after presenting with pancytopenia and splenomegaly. Flow cytometry immunophenotyping identified a scattered (clonal) B-cell population compatible with HCL. Abnormal B-cells were characterized as CD45+, CD5−, CD10−, CD11c+, CD19+, CD20+(BRIGHT), CD22+(BRIGHT), CD23−, CD25+, CD38−, CD103+, FMC7+, HLA-DR+, and SIG KAPPA+. Bone marrow smears showed atypical lymphoid spicules with inconspicuous nucleolei. Core biopsy showed hypercellularity along with patchy-mononuclear hairy cell infiltrate, with mostly round nuclei, spaced out with ample clear cytoplasm, demonstrating strong annexin A1 immunostaining. Treatment for HCL commenced in August 2018 with a continuous intravenous infusion of cladribine at 0.1 mg/kg/day for 7 days.^2^ An abdominal ultrasound performed in September 2018 revealed mild splenic enlargement, followed by repeat bone marrow biopsies in October 2018 and January 2019, showing signs of stable and, progressive disease, respectively. Therefore, the patient was switched to a regimen of pentostatin. Although the complete response rate is lower with each subsequent line of therapy among purine analogs (PNAs), patients can still be successfully re-treated with cladribine or pentostatin.^3^ After six cycles, the patient achieved remission with a very low monoclonal B-cell population (0.1%). Almost a year after the last pentostatin cycle, laboratory testing revealed a white blood cell count of 2.1 × 10^9^/L, absolute neutrophilic count of 1600 cells/mm^3^, hemoglobin 12.9 g/dL, and platelet count of 53 × 10^9^/L. Flow cytometry and peripheral blood smears showed persistently low levels of CD20+, CD103+, CD11c+, and CD25− monoclonal B-cell populations (2%). His disease had relapsed, and moxetumomab pasudotox was started as a second-line salvage therapy. He was given six cycles over 28 days, administered at 0.04 mg/kg every other day.

**Figure 1:**
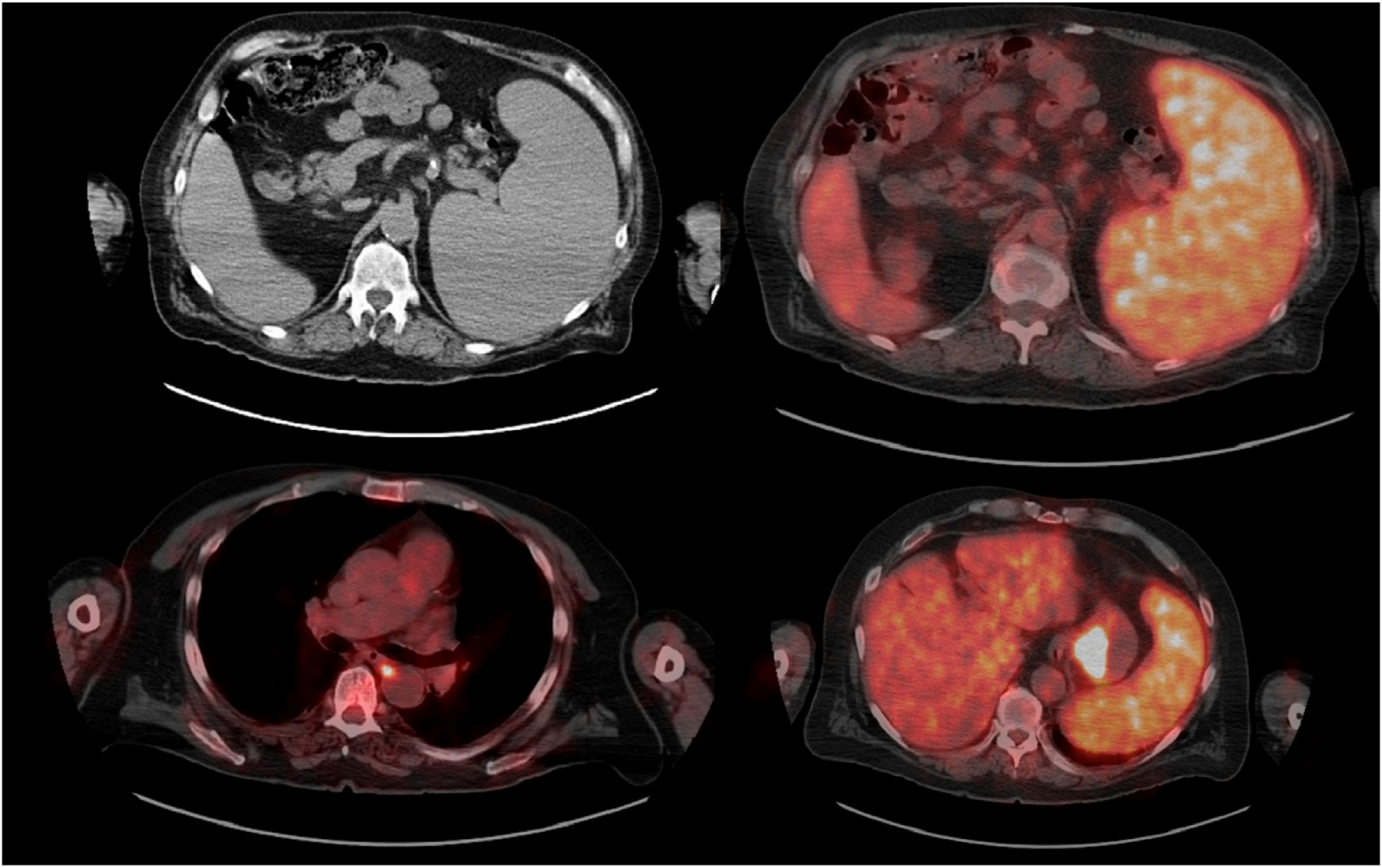
Massive hepatosplenomegaly along with hypermetabolic foci seen on positron emission tomography/computed tomography scan in the proximal stomach (standardized uptake value [SUV], 14.0) and between the esophagus and descending aorta (SUV 4.9).

The patient developed a cystic lesion on his back within days of receiving the fourth cycle of moxetumomab. Biopsy revealed moderately differentiated adenocarcinoma. This prompted further testing, and upper gastrointestinal (GI) endoscopy revealed a gastric mass. A pale lesion was observed in the gastric cardia; this mass had irregular margins and was depressed. Histopathological evaluation confirmed the diagnosis of well-differentiated adenocarcinoma of the intestinal type; helicobacter pylori test results were negative. The patient was a non-smoker and denied alcohol consumption. Ultrasonography revealed that the spleen was markedly enlarged (craniocaudal length, 25 cm). A positron emission tomography/computed tomography (PET/CT) scan showed hyper-metabolic foci in the proximal stomach and between the esophagus and descending aorta, with standardized uptake values of 14.0 and 4.9, respectively, the latter, likely pointing out a metastatic lymph node [Figure 1]. These PET/CT findings of the stomach mass and associated lymphadenopathy were not present on a previous CT scan performed 6 months previously. The mass had a high tumor burden and increased PDL-1 expression. Mutations in PIK23CA, BRCA-2, Rb-1, IGF1R, APC, and PH15 were also observed, which confirmed the diagnosis of stage IV gastric cancer. However, this was not accompanied by GI signs or symptoms. Moreover, the patient had no significant family history of gastric cancer. Past history included diabetes mellitus, hypertension, and osteoarthritis.

Moxetumomab also failed to induce remission, and the flow cytometry results demonstrated an abnormally high monoclonal B-cell population (21%) comprising CD11c+, CD19+, CD20+(BRIGHT), CD22+(BRIGHT), CD45+, CD25+, CD103+, FMC7+, HLA-DR+, and SIG KAPPA + cells.

Systemic therapy was continued, and a referral for radiation therapy was made. A total of 45 Gy/25 Fx was administered to the hypermetabolic foci. The plan was to follow up with a repeat PET/CT scan; however, the patient's condition deteriorated, which ultimately led to death.

## Discussion

Patients with HCL are inherently prone to secondary malignancies. Risk factors appear to be more closely related to the HCL tumor burden than to genetic predisposition or treatment effects. The increased risk of secondary cancers in patients with HCL may potentially be associated with immunosuppression (pre-existing or disease-related) or with the treatment of HCL. Although improved treatment options may allow more time for secondary cancers to arise in the aging population, this is debatable.^4^ Treatment with interferon, previously believed to be associated with an increased residual risk of a second neoplasm,^4^ did not exert an oncogenic effect in 1022 patients with adequate follow-up in another trial.^5^ Although some studies suggest that the development of secondary cancers, such as genito-urinary tumors, may be possible in certain patient groups,^6^ others have shown no causality.^7^

A cohort of 279 patients with HCL was analyzed over a 10-year period, revealing that cladribine/pentostatin was the best choice throughout the study. The use of first-line PNAs led to maximal numbers of complete responses, lowest cumulative incidences of relapse (CIR), longest therapy response, and prolonged median relapse-free survival.^8^ PNAs also do not pose any significant carcinogenic risk.^8^ It is still largely unknown whether the number of cycles has any influence on the outcomes and/or leads to secondary cancers.^8^

Although moxetumomab pasudotox has been discontinued for chronic lymphocytic leukemia, precursor cell lymphoblastic leukemia, and non-Hodgkin's lymphoma,^9^ it remains the most successful recombinant immunotoxin that can induce complete remission following recurrent HCL relapse.^10^ A phase 1 trial led to complete remission in approximately 57% of refractory/relapse cases. More importantly, most of these cases also showed simultaneous removal of minimal residual disease (MRD), which ultimately prolonged the complete remission period.^11^ The cut-off for determining the presence of remnant MRD has been estimated to be ≥ 0.01% of HCL cells using bone marrow aspirate flow cytometry.^12^ This means that HCL relapses, especially in younger patients, can now be managed with better outcomes.

Although moxetumomab pasudotox has shown promising results, it has important side effects, which can be classified as treatment-emergent adverse events (TEAEs) and treatment-emergent serious adverse events (TESAEs). A pivotal phase 3 clinical trial conducted at 32 centers across 14 nations concluded that approximately 96% of the patient population was affected/at risk of TEAEs, whereas 35% were affected or at risk of developing TESAEs. The proportion of patients who did not complete the trial due to AEs, withdrawal, lack of response, and further progression amounted to 25%, whereas those who shifted to other therapeutic agents were slightly above 20%. Of all the severe adverse reactions requiring discontinuation of treatment and possible reconsideration, hemolytic uremic syndrome (HUS), vascular/capillary leak syndrome (CLS), and renal toxicity are the most notable.^13^ However, patients who no longer respond to alternative treatment modalities or those who are unable to tolerate further treatment with PNAs due to myelosuppression and/or subsequent infections, such as older adults, may be considered suitable candidates for this agent. The safety profile of moxetumomab pasudotox has been established, and it is recommended to continue treatment for up to six cycles unless there is disease progression or the occurrence of severe toxicity events such as CLS or HUS. Moxetumomab pasudotox should be used with caution in ongoing HUS, hypocalcemia, severe anemia, markedly decreased hepatic and/or renal function, as well as in pregnancy.^13^

The metabolism of moxetumomab pasudotox remains largely unknown. However, it is proposed that the drug undergoes proteolytic degradation into smaller peptides/amino acids.^9^ Moreover, the probability of long-term side effects may have been underestimated. Attribution to secondary malignancies is also questionable because our patient acutely developed a second cancer that, to the best of our knowledge, could not be explained by the sequence of events. Our patient did not have any apparent risk factors for gastric cancer, such as smoking, alcohol, *Helicobacter pylori* infection, obesity, or excessive consumption of salt/N-nitroso-containing foods.^14^ Moreover, there were no specific findings indicating a gastric malignancy on PET/CT. We believe this case report demonstrates the first known association between HCL treatment with moxetumomab pasudotox and the development of an advanced gastric adenocarcinoma. Our patient had completed all cycles of moxetumomab before the second cancer diagnosis; hence, HCL treatment was continued. Halting chemo/immunotherapy abruptly may lead to increased tumor burden and metastatic potential, allowing more time for cancerous cells to proliferate.^15^

Long-term and rare side effects of moxetumomab still remain undocumented. Thus, physicians should remain vigilant when prescribing moxetumomab.

## Funding

None.

## Author contributions

Shahan Tariq and Muhammad Ammar Bin Hamid: Literature review, critical analysis, follow-up of clinical cases, and manuscript writing. Nazi Rahman, Lindsey Oleary, and Kristine Wong: Critical revision and proofreading. Aasim Sehbai: Conceptualization and supervision.

## Ethics statement

All procedures performed in this study involving human participants were in accordance with the ethical standards of the institutional and/or national research committee and with the 1964 *Declaration of Helsinki* and its later amendments or comparable ethical standards. Informed consent was obtained from the patient included in the study.

## Data availability statement

The datasets used in this study are available from the corresponding author upon reasonable request.

## Conflicts of interest

None.
